# Overlooked and misunderstood: can glutathione conjugates be clues to understanding plant glutathione transferases?

**DOI:** 10.1098/rstb.2023.0365

**Published:** 2024-09-30

**Authors:** Nikola Micic, Asta Holmelund Rønager, Mette Sørensen, Nanna Bjarnholt

**Affiliations:** ^1^ Department of Plant and Environmental Sciences, University of Copenhagen, Frederiksberg 1871, Denmark; ^2^ Copenhagen Plant Science Center, University of Copenhagen, Frederiksberg 1871, Denmark; ^3^ Novo Nordisk Pharmatech A/S, Køge 4600, Denmark

**Keywords:** glutathione conjugate, GST function, glutathione transferase, GST, plant GST, plant metabolism

## Abstract

Plant glutathione transferases (GSTs) constitute a large and diverse family of enzymes that are involved in plant stress response, metabolism and defence, yet their physiological functions remain largely elusive. Consistent with the traditional view on GSTs across organisms as detoxification enzymes, *in vitro* most plant GSTs catalyse glutathionylation, conjugation of the tripeptide glutathione (GSH; γ-Glu-Cys-Gly) onto reactive molecules. However, when it comes to elucidating GST functions, it remains a key challenge that the endogenous plant glutathione conjugates (GS-conjugates) that would result from such glutathionylation reactions are rarely reported. Furthermore, GSTs often display high substrate promiscuity, and their proposed substrates are prone to spontaneous chemical reactions with GSH; hence, single-gene knockouts rarely provide clear chemotypes or phenotypes. In a few cases, GS-conjugates are demonstrated to be biosynthetic intermediates that are rapidly further metabolized towards a pathway end product, explaining their low abundance and rare detection. In this review, we summarize the current knowledge of plant GST functions and how and possibly why evolution has resulted in a broad and extensive expansion of the plant GST family. Finally, we demonstrate that endogenous GS-conjugates are more prevalent in plants than assumed and suggest they are overlooked as clues towards the identification of plant GST functions.

This article is part of the theme issue ‘The evolution of plant metabolism’.

## 1. Introduction

Glutathione transferases (GSTs) are a superfamily of enzymes that are most well-known for the activity they were named by: catalysing the conjugation of the reduced form of the tripeptide glutathione (GSH; γ-Glu-Cys-Gly) onto molecules with an electrophilic centre in their structure. This conjugation, also known as glutathionylation, results in the formation of glutathione conjugates (GS-conjugates) [1]. As a diverse class of enzymes, GSTs are found across plants, animals and microbes [1–3], with plants harbouring the highest numbers by far, reflecting the importance of GSTs in plant evolution and adaption of their metabolism to withstand numerous stresses and changing environments. However, the majority of their physiological functions remain a mystery. In most cases where a specific role or effect has been assigned to a GST, the exact mechanism is unresolved [4,5]. In all organisms, GSTs are present that act as detoxification enzymes responsible for inactivating harmful and toxic xenobiotics of synthetic and natural origin by glutathionylation [6]. In plants, this is most well-known and described for herbicides [4,6], but GSTs also catalyse such detoxification of pathogenic toxins and endogenous toxic compounds produced upon stress exposure [7,8]. In some cases, GSTs are known to take part in small-molecule biosynthesis, and here their initial recruitment to the biosynthetic pathways may often be explained by the need to detoxify a novel autotoxic intermediate or breakdown product resulting from diversification of the original pathway [9–12]. Compared to animals in which GSTs also partake in xenobiotic detoxification and stress response, the larger numbers of plant GSTs imply that they have additional functions, especially as some plant GSTs exhibit little or no glutathionylating activity [13]. In addition, the existence of multiple evolutionarily distinct plant GST classes and upregulation of *GST*s during different developmental stages in stress-free conditions implies that plant GSTs are part of several different endogenous processes, including some unrelated to detoxification or stress response [14,15].

Although the roles of GSTs in plants appear to involve a broad repertoire of catalytic as well as non-catalytic activities, as pointed out in previous reviews [4,16–18], glutathionylating activity has been readily confirmed for numerous plant GSTs in *in vitro* assays, using different synthetic or natural substrates. On the other hand, GST-catalysed production of endogenous GS-conjugates is rarely confirmed *in vivo*, as outlined in this review. This may, however, be partly explained by the transient nature of endogenous GS-conjugates, causing them to accumulate in extremely low amounts or not at all [19–21], as discussed in this review. Discovery and identification of endogenous GS-conjugates, in combination with transcriptomics, targeted gene mutations, stress induction studies or enzymatic studies [11,21–23] has indeed proven a valuable tool in deciphering the roles of GSTs [11,21–23]. This review therefore highlights the status and recent developments in plant GS-conjugates and their possible related GST functions, seen in the light of the evolution of the plant GST enzyme family.

## 2. The evolutionary road towards glutathione transferase functional diversification

The GST protein family is one of the largest in plants with up to approximately 100 members in diploid plant species (table 1) compared to 16 in humans [41]. It is a common assumption that the phenotypic and genomic diversification of plants is a direct result of their remarkable ability to evolve at high rates through various gene duplication events, and half of all genes in flowering plants are estimated to have a duplicate copy [42–45]. Accordingly, duplication events are thought to have led to the emergence and expansion of large plant families such as the cytochrome P450 family (reviewed elsewhere in the current special issue), the subtilase family and the GST family [46–48]. The enzyme families consist of multiple genes, originating from a single duplication event, with highly similar biochemical functions [49]; in the case of GSTs, the common ancestor is thought to be thioredoxin, the basic structure of which is conserved in the GSH-binding G-site across classes [50]. This site, located in the N-terminal thioredoxin-like fold domain, is highly conserved across different GST classes and allows the binding of GSH through a series of hydrogen bonds that stabilize the GSH molecule in its extended conformation [18]. Located in the C-terminal region, the H-site on the other hand is characterized by a highly variable sequence and structure leading to its high conformational flexibility, allowing binding of various co-substrates with different degrees of specificity and frequently causing a high substrate promiscuity in GSTs [6,18].

**Table 1.** Overview of identified GST genes and classes in various plant species. All listed species are diploid, with the exception of *Brassica napus* (allotetraploid) and *Physcomitrella patens* (dominantly haploid). *Physcomitrella patens*, spreading earthmoss; *Larix kaempferi*, Japanese larch; *Sorghum bicolor*, sorghum; *Setaria italica*, foxtail millet; *Oryza sativa*, Asian rice; *Camellia sinensis*, tea plant; *Gossypium raimondii*, Peruvian cotton; *Gossypium arboretum*, tree cotton; *Medicago truncatula*, barrelclover; *Glycine max*, soybean; *Malus domestica*, apple; *Pyrus communis*, common pear; *Capsella rubella*, pink shepherd’s purse; *Brassica oleracea*, cabbages; *Brassica napus*, rapeseed; *Arabidopsis thaliana*, arabidopsis; *Populus trichocarpa*, black cottonwood; *Cucurbita maxima*, winter squash.

species (family)	total	DHAR	lambda	phi	tau	TCHQD	theta	zeta	other cys-GSTs^c^	other GSTs	ref.
**Bryophyta**											
*Physcomitrella patens* (Bryopsida)	**37**	3	1	10	—	5	3	1	9	5	[24]
**Gymnospermae**											
*Larix kaempferi* ^a^ (Pinopsida)	**27**	1	3	7	11	1	1	2	—	1	[25]
**Angiospermae**											
*Sorghum bicolor* (Poaceae)	**93**	3	4	18	55	1	2	4	3	3	[15]
*Setaria italica* ^a^ (Poaceae)	**73**	2	6	18	44	1	—	1	—	1	[26]
*Oryza sativa* ^a^ (Poaceae)	**82**	2	3	17	52	1	1	4	—	2	[27]
*Camellia sinensis* (Theaceae)	**88**	—	10	4	61	—	5	—	—	8	[28]
*Gossypium raimondii* (Malvaceae)	**59**	3	3	7	38	1	3	2	—	2	[29]
*Gossypium arboreum* (Malvaceae)	**49**	3	3	6	29	1	3	2	—	2	[29]
*Medicago truncatula* (Fabaceae)	**73**	2	4	9	49	1	3	2	—	3	[30]
*Glycine max* (Fabaceae)	**101**	4	7	14	63	3	3	3	—	4	[31]
*Malus domestica* (Rosaceae)	**52**	3	1	14	33	1	—	—	—	—	[32]
*Pyrus communis* ^a^ (Rosaceae)	**57**	5	4	11	24	1	1	3	3	5	[33]
*Capsella rubella* (Brassicaceae)	**49**	3	2	12	25	1	1	3	—	2	[34]
*Brassica oleracea* ^a,b^ (Brassicaceae)	**65 (63)**	4	3	14	28	1	2	2	6	5 (3)	[35]
*Brassica napus* ^b^ (Brassicaceae)	**179 (170)**	9	5	39	84	2	4	6	13	17 (11)	[36]
*Arabidopsis thaliana* (Brassicaceae)	**53**	4	3	13	28	1	2	2	—	—	[37,38]
*Populus trichocarpa* (Salicaceae)	**81**	3	3	9	58	1	2	2	—	3	[39]
*Cucurbita maxima* ^a^ (Cucurbitaceae)	**32**	—	1	3	18	—	2	3	2	3	[40]

^a^ BLAST search to identify GSTs and/or the phylogenetic tree was carried out using GST sequences from a single species.

^b^ The presence of both a GST N- and C-terminal domain has not been verified in all sequences. The number in brackets indicates the number of sequences within the specific class(es) which were demonstrated to contain both an N- and a C-terminal domain.

^c^ Total number of GST genes classified as one of the four additional GST classes containing a cysteine in their active site (GSTI, GHR, GSTH or mPGES-2).

To date, more than 30 phylogenetic studies on plant GST families have been published, from species ranging from mosses to trees (table 1 and electronic supplementary material, table S1), and 14 GST classes have been identified (see below). Although such studies can and do provide interesting insights into the structure and evolution of the enzyme family, the field suffers from a lack of a standardized pipeline for identifying and annotating GST-encoding genes in plant genomes. Commonly, GST-encoding genes are identified by a BLASTP search against a genome, proteome or transcriptome. The latter two are the only option when a species lacks a reference genome, but then target only enzymes that happen to be expressed in certain tissues or under specific conditions and may lead to incomplete results [51–56]. Furthermore, although BLAST searches using GST sequences from other organisms have repeatedly led to the identification of new sub-classes [24,37,57], in most publications the GSTome was identified using only plant sequences as queries, sometimes originating from a single species, with arabidopsis being a common choice. This approach may limit the identification of GSTs as some of the classes identified in plants are only found in the earliest evolved plants, and the arabidopsis genome, for instance, encodes 53 GSTs but only seven of the 14 classes [37,38]. Also, with the continuous advancement of bioinformatic and genomic tools and because the attention to the GST family is recent, many smaller GST classes were only discovered within the last 10–15 years and hence do not feature in earlier studies. Furthermore, due to the highly variable H-site, sequence identity is low between classes. Consequently, BLASTP approaches using only the GSTome of arabidopsis and/or maybe one other species might lead to an incomplete result. In addition, there is no complete consensus regarding which characteristics define a GST. While partial *GST* fragments are usually believed to be non-expressed pseudogenes [24,31,34,39], some studies include such sequences in the total count of GSTs in a species, in particular sequences containing only the N-terminal part of the enzymes [58–68]. As none of these partial sequences has been confirmed to be expressed or functionally characterized neither *in vitro* nor *in vivo*, it remains to be shown whether they arose from genome annotation errors, are pseudogenes or actually have some until now undiscovered function. Finally, in other instances, phylogenetic analysis and subsequent classification are performed using only the highly conserved N-terminal part of the sequences, whereas the classical classifications and also most GSTome studies are based on the alignment of the entire protein sequences. Such differing approaches to the phylogenetic analyses may lead to incompatible results; there are indeed examples where phylogenetic analysis based on N-terminal sequences alone led to class annotations of sequences that could not be confirmed by the presence of class signature motifs beyond the overall conserved N-terminal [21]. In order to enable comparison and overall conclusions across studies and species, in our view, a GSTome should meet the following minimal set of standards: the BLAST search or other sequence identification should (1) be performed against a genome rather than transcriptome or proteome, (2) use query sequences from more than one species, that (3) cover all 14 identified plant GST classes. Ideally, the searches should also use bait sequences from other kingdoms, but this is not critical. Furthermore, all sequences should (4) contain both GST N- and C-termini to form putative fully functional enzymes with G- and H-sites. Furthermore, the phylogenetic analysis should follow standard guidelines, such as classifications being supported by overall bootstrap values >70 and should only include GST classes from the plant kingdom. In table 1, we list the published GSTome studies that meet criteria 1–4 and have strongly supported classifications, and their results in terms of the number of members of each class. We include all known classes but have omitted studies where the number of GSTs within classes or species might be imprecise due to deviation from one or more of the criteria mentioned above. Two of the studies listed in table 1, *Brassica oleracea* and *Brassica napus*, have included genes lacking a C-terminal domain in their GSTomes but classified them independently, enabling us to subtract them from the total count, and the species are therefore included in the table. The studies omitted from table 1 are instead shown in electronic supplementary material, table S1, since they do contribute to the total body of knowledge about plant GSTs, as the overall trends within total number of GSTs and division between classes support the results collected in table 1.

The 14 GST classes identified in plants are as follows: tau (GSTU), phi (GSTF), theta (GSTT), zeta (GSTZ), lambda (GSTL), hemerythrin (GSTH), iota (GSTI), dehydroascorbate reductase (DHAR), glutathionyl-hydroquinone reductase (GHR), tetrachloro-hydroquinone dehalogenase (TCHQD), metaxin, Ure2p, γ-subunit of the eukaryotic translation elongation factor 1B (EF1Bγ) and microsomal prostaglandin E synthase type 2 (mPGES-2) [5,16,18]. GST classes GSTU, GSTL and DHAR are plant specific with GSTF and GSTU being by far the biggest [1]. The GSTF class was considered to be plant specific, but proteins with sequences similar to plant GSTFs were recently also found in bacteria, fungi and protists [69,70]. As pointed out in most plant GSTome publications, table 1 shows that the GSTF and GSTU classes have the highest number of members overall, with the GSTU class typically being the largest but also the most variable in size. For instance, in the monocot sorghum, the GSTUs constitute roughly 59% of all GST genes (55/93), 71% in black cottonwood (58/81) and 64% in the cotton species *Gossypium raimondii* (38/59; table 1). Most plant GSTs act as homodimers or heterodimers [71–73], and dimerization through interactions of C- and N-terminals of monomers is typical for all dimeric GSTs, with the exception of GHRs, which dimerize upon the interaction of C-terminals of monomers [5]. The lambda and DHAR classes are known exceptions acting as monomeric proteins [1], but only a small number of GSTs belonging to GSTF, GSTU, GSTZ and GSTL classes have solved crystallographic structures [16,74–76]. The active site of GSTs is located in the conserved G-site close to the N-terminal, and depending on the catalytic active site amino acid, plant GSTs are divided into two overall groups with the GSTT, GSTZ, GSTF, GSTU and TCHQD classes generally containing a conserved serine (Ser) residue [1], while the catalytic residue in members of the GSTL, GSTI, GSTH, DHAR, GHR, mPGES-2 and metaxin classes is a cysteine (Cys) [5]. The exact catalytic residues of EF1Bγ and Ure2p GSTs are unknown [5]. The catalytic activity is generally accepted to be dictated by these conserved catalytic residues, with Ser-GSTs catalysing the conjugation of GSH onto a reactive molecule [16], while Cys-GSTs remove GSH from GS-conjugates in a reaction known as reductive deglutathionylation [5]. This has been confirmed for several members of each of the Ser-GST classes [13,77,78], whereas deglutathionylating activity has been confirmed for the GSTL and GHR classes [5] and, in some cases, unusual GSTFs that have Cys in their active site [78]. The latter belongs to a subgroup of GSTs identified as responsible for the most well-documented non-catalytic GST function, the ligandin binding of anthocyanins in several plant species that facilitates their transport into the vacuoles where they are stored [14,79–81].

It is believed that GST-mediated transport of the reactive anthocyanins across the cytosol protects them from degradation [82]. The majority of the anthocyanin-binding GSTs belong to the otherwise glutathionylating Ser-GSTs in the GSTF class; however, their active site amino acid is a Cys [75,83]. While they are traditionally described as not having a catalytic function in anthocyanin biosynthesis, *Pt*GSTF8 from black cottonwood (*Populus trichocarpa*) was recently demonstrated to catalyse a dehydration reaction in the anthocyanin biosynthetic pathway [83]. The proposed reaction mechanism does not proceed via formation or cleavage of a GS-conjugate but does involve GS^−^ participating in a transient hydrogen bond with first the cyanidin substrate and then the catalytic Cys-residue. A Cys-Ser mutation did not abolish the activity; however, it caused the enzyme to be insensitive to oxidation [83]. Possibly, the apparent evolutionary advantage that led to the catalytic residue change in the subclass of anthocyanin-related GSTFs is caused by either the gain of a capacity for redox regulation of the new enzymatic activity or the loss of the glutathionylating activity, preventing unwanted reactions.

Functional divergence among the Ser-GSTs is also seen for arabidopsis zeta GST (*At*GSTZ1), which acts as a maleylacetone isomerase similar to mammalian GSTZs that catalyse isomerization of maleylacetoacetate to fumarylacetoacetate in the tyrosine degradation pathway [84]. Sequence similarities and gene upregulation during degradation of aromatic amino acids suggest that this may be a conserved function among GSTZs [85]. The proposed mechanism proceeds via a transient glutathionylation–deglutathionylation cycle [77,85]. In other words, in this case, a Ser-GST performs both reactions, whereas in the pentachlorophenol-degrading bacterium *Sphingobium chlorophenolicum*, a Cys-GST creates a similar reaction cycle [29]. These findings challenge the understanding that the enzyme function is solely dictated by the catalytic amino acid.

Across plant genomes, *GST*s are found in non-random uneven chromosomal distribution and in many clusters that appear to originate from segmental or tandem duplication events, with the clusters most commonly harbouring GSTFs and GSTUs, possibly caused by multiple gene duplication events within these two classes. The GSTUs are so far not found in moss and green algae species, and it is commonly assumed that the expansion of the GSTU and GSTF classes is the result of evolution driven by requirements for functional diversification to counteract the multiple sources of biotic and abiotic stress associated with the expansion of land plant habitats [24,34]. As such, the duplicated genes that have been retained in the genomes are expected to have evolved under purifying selection [39,40,86,87] undergoing neo- or sub-functionalization. This seems to be the case for the anthocyanin-related GSTs that are for the large part highly homologous in sequence and have an apparently advantageous active site amino acid substitution. Overall, the C-terminal appears to have evolved under more relaxed constraints compared to the N-terminal, leading to a diverse range of co-substrate specificities and novel GST classes while conserving the ancestral function. Within classes, duplicated genes may evolve to obtain variations in *cis*-acting elements, promotor regions or introns, resulting in different expression patterns of otherwise catalytically similar proteins [26,30]. This was demonstrated by He *et al*. [34], who investigated seven tandem-arrayed genomic clusters in *Capsella rubella* containing GSTU and GSTF encoding sequences that were found to be highly diversified in terms of expression patterns, enzymatic activities and substrate specificities [34]. Similar diversifications within the *GSTU*, *GSTF* and *GSTL* classes have been reported from several other species [24,36,39,75,86,88,89]. In addition to the variation between classes in protein structure, in particular of the poorly conserved H-site, and catalytic amino acids, they also differ in their inducibility and subcellular localization [17,80,90]. While most GSTs have been found to be cytosolic proteins [13], some are localized to chloroplasts, peroxisomes, nuclei or the plasma membrane [13,75,91], demonstrating the high degree of evolution and presumed sub- and neo-functionalization GSTs of all classes have undergone. To this date, the catalytic function of only a small fraction of GSTs has been elucidated, and multiple classes remain entirely uncharacterized, leaving much further investigation before we fully understand the functional and evolutionary diversification of the GST family.

## 3. Glutathione-conjugates: potential clues towards identification of glutathione transferase activities

The only plant GST function involving endogenous metabolites that has been found repeatedly across species is the anthocyanin transport, which does not involve the formation of GS-conjugates. Even this apparently conserved functionality is partly puzzling as one of the classical enzymes in this group, *Zm*BZ2 (Bronze2 from maize), does not cluster clearly with any GST class in published phylogenetic trees [92,93]. The newly discovered function in this group of enzymes is enzymatic but proposedly does not involve glutathionylation or deglutathionylation [83]. The DHAR and tyrosine-degrading GSTZ activities demonstrated in arabidopsis are also assumed to be conserved between species due to the conserved nature of the substrates and pathways and do involve the formation of GS-conjugates, but only as catalytic cycle intermediates that are never released. So, are GS-conjugates really clues in the search for GST functions beyond detoxification, and if yes, why have we not come any further? The following sections shed light on this question and summarize the current knowledge on GS-conjugates and their potential, or in some cases even identified, GST partners.

**Figure 1.**
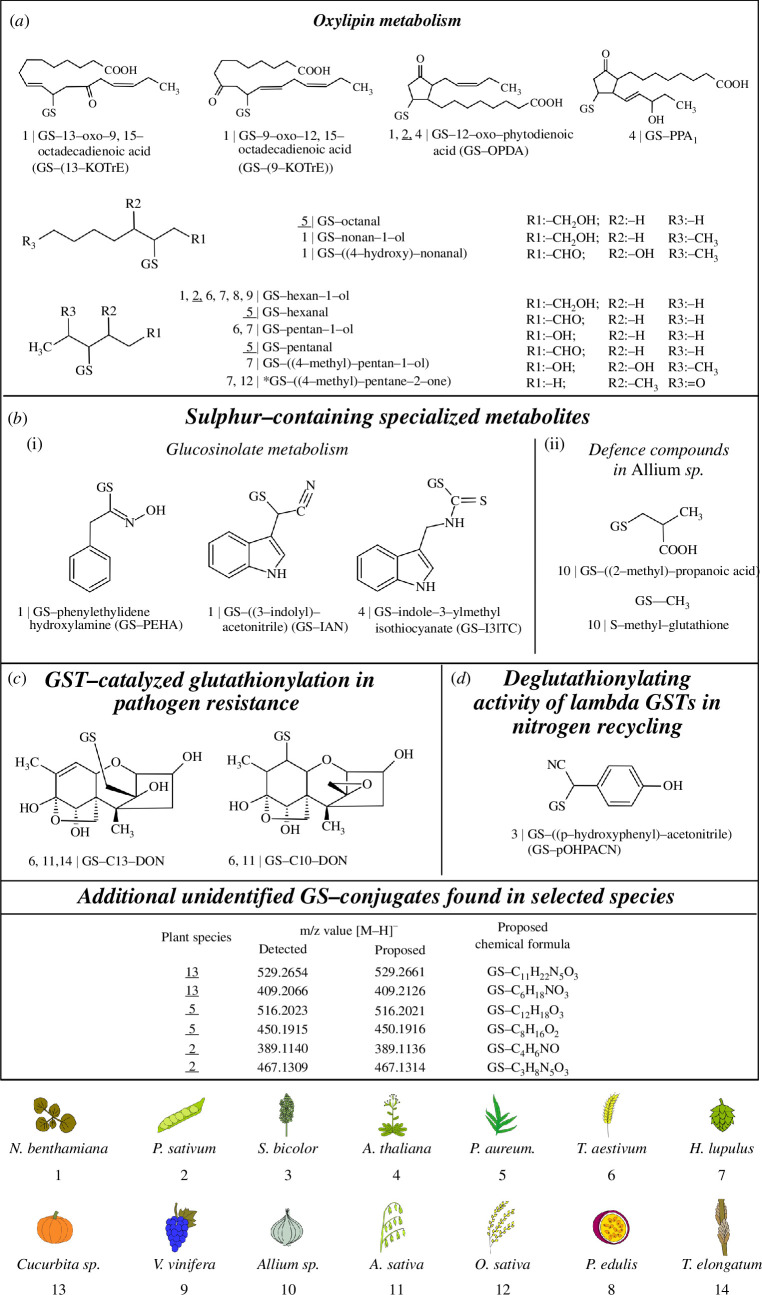
Endogenous GS-conjugates found in different plant species. Identified GS-conjugates are arranged in groups according to their specific metabolic origins in the plants. The number on the left side of the conjugate name indicates in which species it was identified. Underlined numbers signify that this is the first report of the given GS-conjugate in the respective plant species. The oxylipin-derived GS-conjugates were identified by comparison with authentic standards produced as described in electronic supplementary material, file S2. Listed at the bottom of the figure, the analyses also led to discoveries of unknown GS-conjugates, as identified by their characteristic fragmentation (electronic supplementary material, file S3) according to Dieckhaus *et al*. [27] and the occurrence of at least four characteristic fragments out of seven known to result from fragmentation of the GS-moiety of the conjugates. *Nicotiana benthamiana*, tobacco; *P. sativum*, green peas; *S. bicolor*, sorghum; *A. thaliana*, arabidopsis; *P. aureum*, bluestar fern; *T. aestivum*, wheat; *H. lupulus*, hops; *Cucurbita* sp., pumpkin; *V. vinifera*, grapevine; *Allium* sp., onion species; *A. sativa*, oat; *O. sativa*, rice; *P. edulis*, passion fruit; *T. elongatum*, wheat grass.

Figure 1 shows the identified structures of GS-conjugates and lists their plants of origin; in addition to the examples from the literature described below, we show results from our ongoing searches for GS-conjugates in different plants. Endogenous GS-conjugates identified in plants to date result from a reaction of the nucleophilic thiol group in GSH with electrophilic centres of different reactive species, i.e. a reaction that can in some cases proceed spontaneously as a chemical reaction in an *in vitro* system [94], as shown in table 2. This makes it difficult to discover or prove glutathionylating activity of a GST in a classical enzyme characterization set-up or even in a knockout plant. Lack of chemotypes in knockout plants may also result from the high substrate promiscuity that many GSTs exhibit [22], possibly because they have dual functions as general detoxifying enzymes that serve additional endogenous purposes. Despite the obvious ubiquitous presence of GS-conjugates across the plant kingdom demonstrated in figure 1, they are still somewhat rarely reported, possibly because they are often present in very low amounts and hence overlooked. Ironically, as explained in the following sections, this low abundance in many cases reflects their status as intermediates in important metabolic pathways, which are quickly further catabolized by other enzymes.

**Table 2.** GS-conjugates identified across different plant species involved in different biological processes (i-iv), and GSTs responsible for their biosynthesis.(i) – production of aroma related compounds; (ii) – regulation of oxylipin-related stress response; (iii) – plant defense; (iv) – nitrogen recovery; *Nicotiana tabacum* – tobacco; *Vitis vinifera* – grapevine; *Humulus lupulus* – hops; *Passiflora edulis* – passion fruit; *Arabidopsis thaliana* – Arabidopsis; *Allium sp.* – onion species; *Thinopyrum elongatum* – tall wheatgrass; *Triticum aestivum* – wheat; *Avena sativa* – oat; *Oryza sativa* – rice; *Sorghum bicolor* – sorghum; GS-(13—KOD(Tr)E) – glutathione conjugate of keto-octadecadi(tri)enoic acid; GS-OPDA – glutathione conjugate of 12-oxo-phytodienoic acid; GS-PPA_1_ – glutathione conjugate of phytoprostane A_1_; GS-PEHA – glutathione conjugate of phenylethylidene hydroxylamine; GS-IAN – glutathione conjugate of indol-3-acetonitrile; GS-I3ITC – glutathione conjugate of indole-3-ylmethylisothiocyanate; ; GS-C10(C13)-DON – glutathione conjugate of deoxynivalenol (C13 and C10 as conjugation sites); GS-VirE2 – glutathione conjugate of VirE2 protein; GS-pOHPACN – glutathione conjugate of p-hydroxyphenylacetonitrile. GS-C10(C13)-DON, glutathione conjugate of deoxynivalenol (C13 and C10 as conjugation sites); GS-IAN, glutathione conjugate of indol-3-acetonitrile; GS-I3ITC, glutathione conjugate of indole-3-ylmethylisothiocyanate; GS-(13-KOD(Tr)E), glutathione conjugate of keto-octadecadi(tri)enoic acid; GS-OPDA, glutathione conjugate of 12-oxo-phytodienoic acid; GS-PEHA, glutathione conjugate of phenylethylidene hydroxylamine; GS-pOHPACN, glutathione conjugate of p-hydroxyphenylacetonitrile; GS-PPA1 , glutathione conjugate of phytoprostane A1 ; GS-VirE2, glutathione conjugate of VirE2 protein.

	GS-conjugate	Plant species	GST candidates^a^	Presence of downstream products^b^	Spontaneous formation	Ref.
(i)	GS-pentan−1-ol	*Humulus lupulus*	—	Yes	Yes	[95]
	GS-((4-methyl)-pentan−1-ol)	*Humulus lupulus*	—	Yes	Yes	[95,96]
	GS-((4-methyl)-pentane−2-one)	*Humulus lupulus*	—	No	Yes	[97]
(i) + (ii)	GS−3-hexan−1-ol	*Nicotiana tabacum*	NtGSTUs, NtGSTFs	Not investigated	Yes	[12]
		*Vitis vinifera*	VvGST3, VvGST4, (VvGST1)	Yes	Yes	[23]
		*Humulus lupulus*	—	Yes	Yes	[93,94]
		*Passiflora edulis*	—	Yes	Yes	[84]
(ii)	GS−3-nonan−1-ol	*Nicotiana tabacum*	NtGSTUs, NtGSTFs	Not investigated	Yes	[12]
	GS-((4-hydroxy)-nonanal)	*Nicotiana tabacum*	NtGSTUs, NtGSTFs	Not investigated	Yes	[12]
	GS-(13-KOD(Tr)E)	*Nicotiana tabacum*	NtGSTUs, NtGSTFs	Not investigated	Yes	[12]
(ii) + (iii)	GS-OPDA	*Arabidopsis thaliana*	AtGSTU6/U10/U17/U19/U25	Not investigated	Yes	[22]
		*Arabidopsis thaliana*	AtGST6	Not investigated	Yes	[89]
		*Nicotiana tabacum*	NtGSTUs, NtGSTFs	Not investigated	Yes	[12]
	GS-PPA_1_	*Arabidopsis thaliana*	AtGST6	Not investigated	Yes^c^	[89]
(iii)	GS-PEHA	*Nicotiana benthamiana*	—	No	Yes	[19]
	GS-IAN	*Arabidopsis thaliana*	AtGSTF6	Yes	Yes	[10,20]
		*Arabidopsis thaliana*	—	Yes	Yes	[98]
	GS-thiohydroxymate (aliphatic)	*Arabidopsis thaliana*	AtGSTF11, AtGSTU20	No	Yes	[99]
	GS-thiohydroxymate (aromatic)	*Arabidopsis thaliana*	AtGSTF9	Yes	Yes	[100]
	GS-I3ITC	*Arabidopsis thaliana*	AtGSTU13	No	Yes	[11]
	GS-((2-methyl)-propanoic acid)	*Allium* sp.	—-	Yes	Yes	[101]
	S-methyl-glutathione	*Allium* sp.	—	Yes	Not investigated	[101]
	GS-C13-DON	*Thinopyrum elongatum*	TeFhb7	No	Yes^d^	[8]
		*Triticum aestivum*	—	Yes	Yes^d^	[102]
		*Avena sativa*				
	GS-C10-DON	*Triticum aestivum*	—	Yes	Yes	[102]
		*Avena sativa*	—			
	GS-VirE2	*Oryza sativa*	OsGSTU5	Not investigated		[7]
(iv)	GS-p-OHPACN	*Sorghum bicolor*	SbGST?	Not investigated	Yes^e^	[21]
			SbGSTL1, SbGSTL2	Not investigated	No	[21]

^a^ Identified through transcriptomics studies on upregulated GST expression, upregulated GST activity through CDNB assay or GST enzyme assay.

^b^ Includes GluCys-conjugates, GlyCys-conjugates, Cys-conjugates and corresponding thiols.

^c^ Specific isomer distribution indicates that GS-PPA1 is produced exclusively by enzymatic reaction in this case.

^d^ Spontaneous formation is possible but amounts are substantially lower compared to presumed *in vivo* enzymatically produced amounts.

^e^ Spontaneous formation of GS-conjugate is possible, however ratio of stereoisomers indicates that formation of GS-conjugate is enzymatically catalyzed.

In the detoxification of GS-conjugates, they are first imported into the vacuole across the tonoplast by ATP-binding cassette (ABC) transporters [103,104] and then degraded in an enzyme-catalysed three-step process, with thiols as final products [105]. In the first reaction, glutamic acid or glycine is cleaved off the GS- part by γ-glutamyl transpeptidases (GGTs) or carboxypeptidases, respectively, followed by further metabolism, first to Cys-conjugates by dipeptidases [105,106] and finally to thiols, catalysed by C-S β-lyases [107]. As described in the next sections, it appears that most of the known endogenous GS-conjugates and the associated GST functions have evolved from an initial requirement for detoxification of reactive endogenous molecules. In some cases, this detoxification has acquired a proposed signalling or regulating function, while in other cases, whole new pathways in specialized metabolism have evolved from the initial GS-conjugation. Table 2 provides an overview of the metabolic processes that are associated with GS-conjugates and specific GSTs to the extent they are known.

## 4. Glutathione transferases as regulators of phytohormone availability

Plant hormones (phytohormones) are a diverse group of biologically active compounds that act as crucial regulators of plant growth and development and of stress responses [108]. Their activities are commonly regulated by conjugation, e.g. the extensively studied glucosylation of gibberellins [95], conjugation of indole-3-acetic acid (auxin) with small aliphatic acids, amino acids or sugars [109] and of jasmonic acid (JA) with amino acids [110]. The compound 12-oxo-phytodienoic acid (OPDA) is a precursor of the well-described phytohormone JA, but OPDA itself also has different and independent phytohormone activities [111]. Glutathionylated OPDA (GS-OPDA) is frequently found in plants (figure 1*a*) [12,22,112], and six different arabidopsis GSTUs are able to catalyse this reaction *in vitro,* suggesting they have a role in the production of GS-OPDA *in vivo* (table 2) [22,113]. Glutathionylation of OPDA is also a spontaneous chemical reaction, which is the likely reason why knocking out a single of these GSTUs (*AtGSTU6*) did not lead to lower GS-OPDA levels in the resulting arabidopsis *gstu6* line [112]. While the exact role of and route to OPDA glutathionylation remains elusive, GS-OPDA has been found to accumulate in the vacuoles of arabidopsis *γ*-glutamyl transpeptidase (*ggt4*) mutant lines that are unable to degrade GS-conjugates. This finding suggests that OPDA glutathionylation serves as a marker for the transport of excess OPDA to the vacuoles for degradation [112], or of OPDA that has escaped the JA biosynthetic pathway and reacted spontaneously with GSH due to the high chemical reactivity of an *α–β* unsaturated bond in its five-carbon ring [94]. JA biosynthesis is a compartmentalized process divided between chloroplasts, where OPDA is produced, and the peroxisome, where it is converted to JA [98]. Hence, OPDA is transported through the cytosol for JA biosynthesis and may escape by dissociation from its potential cytosolic transporter, leading to spontaneous or enzymatic formation of GS-OPDA. However, considering the biological activities and importance of both OPDA and JA, their pathways can be expected to be tightly regulated and other plant species than arabidopsis may have evolved the ability to use OPDA glutathionylation as a regulatory mechanism, with associated GSTs enabling faster and tighter regulation of free, biologically active OPDA. Also, considering that the anthocyanin-transporting GSTs have specifically evolved to *not* glutathionylate the anthocyanins and as several other GSTs have been shown to bind several other ligands such as the structurally different indole compounds (indole-3-acetaldehyde and camalexin), polyphenols (quercitrin and quercetin) [100] and porphyrins [99], it is puzzling if a similar solution has not evolved in the case of OPDA, and such functions may also eventually be discovered beyond arabidopsis. Plants also produce compounds known as phytoprostanes (PPA) with structures similar to OPDA (see example in figure 1*a*). Although their exact functions are unknown, they have signalling properties overlapping with OPDA and one, PPA_1_, was demonstrated to also be a substrate of a GST that was at the time named *At*GST6 [113]. In this study, GS-PPA_1_ and GS-OPDA were both detected in unchallenged arabidopsis plants and found to increase dramatically upon infection with the bacterial pathogen *Pseudomonas syringae*. Interestingly, the relative amount of GS-PPA_1_ was higher than GS-OPDA under both conditions, while the opposite was the case for the unconjugated molecules, indicating that the PPA_1_-glutathionylation was enzymatically controlled [113].

## 5. Glutathionylation in oxylipin detoxification and conversions to other biologically active molecules

Both OPDA, JA and the phytoprostanes belong to the group of compounds known as oxylipins. Generated through the oxidation of membrane lipids or free fatty acids, oxylipins are important indicators of stress in plants, as well as regulators of growth, development and responses to different biotic and abiotic stresses such as wounding, osmotic stress and dehydration, cold and heat stress, ozone and presence of heavy metals [114,115]. Their further metabolism can also yield different types of biologically active oxylipin-derived compounds, possessing either beneficial or harmful activity. Tobacco (*Nicotiana tabacum*) plants exposed to stress (cryptogein-elicited leaves) accumulate the GS-conjugated keto fatty acids shown in figure 1*a* [12]. Although most keto fatty acids can react spontaneously with GSH, the detection of GS-conjugates coincided with increased GST activity (table 2) [12]. Since no single GST has been directly linked to glutathionylation of keto fatty acids, it is possible that stress induces multiple GSTs with this activity to rapidly neutralize the potentially harmful reactive compounds [12].

Keto fatty acids are also processed into different short-chain *α–β* unsaturated aldehydes and alcohols, some of them belonging to the group of compounds known as green leaf volatiles (GLVs) that are important regulatory signals in stress response and communication between plants [116]. GLV-derived GS-conjugates are prevalent in plants (figure 1*a*), with one of the most abundant being GS-3-hexanol, found in tobacco [12], grapevine (*Vitis vinifera*) [23], hops (*Humulus lupulus*) [117,118] and passion fruit (*Passiflora edulis*; figure 1*a*) [106]. In tobacco and grapevine [12,23], increased production of GS-3-hexanol is accompanied by increased production of its precursor *trans*-2-hexenal, an important signal in plant communication and stress response [119]. Increased transcription of tobacco *Nb*GSTUs and *Nb*GSTFs [12] and grapevine *Vv*GST1, *Vv*GST3 and *Vv*GST4 can be linked with glutathionylation of *trans*-2-hexenal and production of GS-3-hexanal, as demonstrated with *Vv*GST3 and *Vv*GST4 having the relevant catalytic activity *in vitro* [23]. The product GS-3-hexanal was subsequently reduced to GS-3-hexanol [12]. Although the exact reason for *trans*-2-hexenal glutathionylation is unknown, it may serve as either (i) signal switch, eliminating the signalling activity of exogenous *trans*-2-hexenal released from other plants in wound responses [116], or (ii) detoxification mechanism for endogenously produced toxic *α−β* unsaturated aldehydes [119,120]. Contrary to glutathionylation of unsaturated aldehydes such as *trans*-2-hexenal, plant uptake of different (un)saturated alcohols is followed by their glycosylation [121], which supports GS-3-hexanol and other GS-alcohols being reduction products of GS-conjugated aldehydes [12]. Glutathionylation of toxic *α−β* unsaturated aldehydes is also seen for *trans*-2-nonenal and 4-hydroxy-2-nonenal, products of membrane lipid degradation [122], and their increased production likewise coincides with increased activity of GSTFs and GSTUs [12]. Although *α−β* unsaturated aldehydes also react spontaneously with GSH, it seems that GST-catalysed glutathionylation is an active response that is regulated through modulation of GST expression and activity in response to increased production of *α−β* unsaturated aldehydes [12].

The presence of GS-conjugates of GLVs in grapevine, hops, passion fruit and also sake rice [101,106,107,117,123] is furthermore related to the production of important thiol aroma compounds in these species, with these compounds being the hypothetical end products of the traditional vacuolar pathway for degradation of GS-conjugates described above [124]. Due to their low odour detection threshold, these volatile thiols can significantly affect the aroma profiles of plant-derived products, well-known from wine and beer [125]. When present in excessive amounts, volatile thiols will lead to the development of repulsive aromas, such as ‘cat urine/sweaty’ aroma sometimes found in Sauvignon Blanc wines, caused by high concentrations of 3-mercaptohexyl acetate, while low concentrations provide pleasant notes of passion fruit/wood [126]. Several different five- and six-carbon thiol aroma compounds are described that appear to be products of GS–GLV conjugation (figure 1*a* and table 2). In several cases, GS-conjugates were shown to be biosynthetic precursors [101,106,117,118], e.g. through detection of the corresponding Cys-conjugates [106,107,117,123], confirming the processing via the GS-conjugate degradation pathway. In grapevine, GS-3-hexanol accumulates in response to biotic and abiotic stress (respectively, *Botryotinia fuckeliana* infection and UV-C irradiation, cold, heat and water deficit) [23], pointing to a role of the conjugate and its proposed associated GSTs in defence or internal signalling, although the volatile thiols may also serve a purpose, for example in the attraction of pollinators. In our work, we found numerous GS–GLVs in unchallenged plants scattered across species (figure 1*a*), pointing to possible roles in developmental signalling rather than defence.

## 6. Sulfur-containing specialized metabolites

Beyond the oxylipin-derived thiols, several sulfur-containing compounds are recognized as important specialized metabolites. Phytoanticipins and phytoalexins such as, respectively, glucosinolates and camalexin, are important defence compounds throughout the Brassicaceae family. Their biosynthesis as well as their further metabolism to other specialized metabolites require the activity of different plant GSTs.

During the biosynthesis of glucosinolates, sulphur is introduced using GSH as sulfur donor, via formation of GS-conjugates [127]. The first evidence of GS-conjugates as biosynthetic intermediates in the biosynthesis of glucosinolates and camalexin was the detection of their precursor GS-conjugates (table 2) that accumulated in arabidopsis knockouts of GS-conjugate catabolizing enzymes γ-glutamylpeptidase-1/-3 (*ggp1*/*ggp3*). Accumulation of GS-conjugates coincided with decreased levels of glucosinolates and camalexin [19,128]. Over-expression of *GGP1/GGP3* led to the opposite scenario [19,20], and in the case of camalexin, the γ-glutamylcysteine- and Cys-derivatives of the precursor GS-IAN (table 2) were also detected [128]. Although it is traditionally assumed that all steps in a biosynthetic pathway are controlled by enzymes, the GS-conjugate intermediates of the glucosinolate and camalexin pathways can also be produced by chemical reactions [96] (table 2). Evolution of the glucosinolate/camalexin biosynthetic pathways led to an autotoxic aci-nitro compound that reacts spontaneously with GSH and is inactivated by this glutathionylation. Hence, this reaction was a prerequisite for plant survival and further evolution of the pathway, and a plant that was able to enhance it by way of GST activity may have had an evolutionary advantage. In the case of glucosinolate biosynthesis, transcriptomics analyses revealed *At*GSTF11/*At*GSTU20 as potential candidates involved in the production of aliphatic glucosinolates [129,130] and *At*GSTF9 as a candidate for the production of indole glucosinolates [96]. However, their over-expression did not result in significant increases in glucosinolate production [19,96,131]. In the case of camalexin biosynthesis, proteome analysis revealed the accumulation of three GST candidates: *At*GSTF2, *At*GSTF6 and *At*GSTF7, and over-expression of mainly *AtGSTF6* led to increased camalexin biosynthesis [10] (table 2). However, the role of *At*GSTF6 in camalexin biosynthesis is unclear as its production was only slightly decreased in *gstf6* knockout mutants [132] and not increased when *At*GSTF6 was co-expressed with the remaining genes in the camalexin biosynthetic pathway in *Nicotiana benthamiana* [132]. The lack of greater effect on glucosinolate/camalexin biosynthesis can be explained by the high chemical reactivity of the aci-nitro precursors and possible functional overlapping between native *N. benthamiana* GSTs and candidate arabidopsis GSTs [19,96,131,132]. Additionally, use of non-natural host systems such as *N. benthamiana* and yeast (*Saccharomyces cerevisiae*) may affect localization of biosynthetic enzymes leading to their lower efficiency [133].

In addition to biosynthesis of glucosinolates, GSTs are involved in the metabolism of their degradation products. Catalysed by myrosinases, hydrolysis of glucosinolates results in the formation of isothiocyanates, compounds with high chemical reactivity and harmful effects on the host plant that can be managed by glutathionylation [17,38,134]. Glutathionylation also acts as a biosynthetic branching point where GS-indol-3-ylmethyl isothiocyanate (GS-I3ITC; figure 1*b*(i)) can be converted into biologically active indol-3-yl-amine (I3A), raphanusamic acid (RA) and 4-O-β-glucosyl-indol-3-ylformamide (4OGlcI3F) or into the phytoalexin brassinin. Co-expression analysis of myrosinase-related genes responsible for indole-3-isothiocyanate (I3ITC) production revealed *At*GSTU13 as a potential candidate responsible for the production of GS-I3ITC [11]. Reduced accumulation of I3A, RA and 4OGlcI3F in the *gstu13* mutant confirmed both involvement of *At*GSTU13 in their biosynthesis and substrate specificity of this enzyme towards indolic isothiocyanates [11]. On the other hand, co-expression analysis failed to identify possible GST candidates involved in brassinin biosynthesis in *Brassica napa*. However, as transient expression of the remaining genes from the brassinin biosynthetic pathway in *N. benthamiana* did not lead to accumulation of glutathionylated I3ITC produced by native GSTs, it is assumed that a specific brassinin biosynthetic GST exists but remains to be identified [135].

*Allium* species also produce sulfur-containing specialized metabolites, with alk(en)ylated cysteine sulfoxides (ACSOs) as main precursors and GS-conjugates as proposed intermediates. Upon tissue disruption, C-S lyases known as alliinases metabolize ACSOs into different volatile disulfides, which act as defence compounds against herbivores and pathogens [136]. Volatile thiols of *Allium* species are also the main constituents of garlic and onion aroma profiles and the compounds that make us cry when we slice onions. The biosynthetic origin of ACSOs has been a matter of discussion since the late 1970s. One proposed biosynthetic pathway is based on detection of labelled GS-conjugates and their downstream products γ-glutamyl peptides and Cys-conjugates upon administration of radiolabelled substrates to garlic and onion plants [97]. The labelled GS-conjugate product (figure 1*b*(ii)) was found exclusively in (*S*)-configuration, implying the involvement of a specific glutathionylating enzyme, responsible for strict stereochemistry [102]. In contrast, in an earlier experiment, administration of ^14^C-valine led to detection of the corresponding Cys-conjugate and no GS-conjugate, leading to the assumption that conjugation takes place with Cys rather than GSH [137]. However, this was also the theory for biosynthesis of glucosinolates and camalexin until the GS-conjugates and GGP enzymes were discovered. In this case, as in others, we now know that the lack of detected GS-conjugates was caused by their rapid further catabolism in the biosynthetic pathways. Given the evidence reviewed here that multiple biosynthetic pathways for sulfur-containing metabolites across plant species appear to have developed from the glutathionylating detoxification pathway, it seems likely that also in the case of the *Allium* thiols, the precursors are GS-conjugates and GSTs are involved (table 2).

## 7. Deglutathionylating activity of lambda glutathione transferases

In the cereal sorghum, biochemical evidence *in planta* strongly supports that two GSTLs catalyse deglutathionylation to convert GS-*p*-hydroxyphenyl acetonitrile (GS-*p*-OHPACN) (figure 1*d*) to free *p*-OHPACN (table 2) in a proposed pathway for recovery of nitrogen from the N-containing defence compound dhurrin [21,138]. Dhurrin is a cyanogenic glucoside, a class of defence compounds that often accumulate in high amounts in young, vulnerable tissues, tying up a large part of the available plant nitrogen. In sorghum, reaction of GSH with dhurrin, leading to formation of GS-*p*-OHPACN, enables recycling of this nitrogen via the subsequent deglutathionylation performed by *Sb*GSTL1 and *Sb*GSTL2 as described above (table 2). As GS-*p*-OHPACN can be produced in a chemical reaction between dhurrin and GSH, it is also in this case still unclear whether a glutathionylating GST is involved. However, factors pointing to this includes stereospecificity of the GSTLs towards one isomer of GS-*p*-OHPACN and co-expression of their encoding genes with the same glutathionylating GSTs in maturing [15] as well as germinating sorghum grain [139].

Deglutathionylating activities towards endogenous plant GS-conjugates (as well as non-endogenous ones) were also demonstrated *in vitro* for three *Ta*GSTLs from wheat (*Triticum aestivum*), three *At*GSTLs from arabidopsis and four *Pt*GSTLs from black cottonwood. In all cases, enzymes showed deglutathionylating activity towards a specific C-ring GS-conjugate of quercetin [75,140]. However, these activities have not been demonstrated *in planta* and deglutathionylation of flavonoids such as quercetin can occur spontaneously [141]. It was therefore proposed that GSTLs catalyse GSH-dependent reduction of reactive toxic quinones that are also produced by oxidative stress-related conditions coinciding with upregulation of GSTL encoding genes [140].

## 8. Glutathione transferase-catalysed glutathionylation in pathogen resistance

Interestingly, although plant GSTs were first discovered as detoxification enzymes of herbicides and serve as xenobiotic detoxification enzymes in other species, and despite the vast arsenal of chemical warfare plants experience from their pathogens, this avenue is scarcely pursued in the search for the identification of endogenous GST functions. However, two examples of plant GSTs, Fhb7 [8] and *Os*GSTU5 [7], contributing to pathogen resistance through detoxification by glutathionylation were recently published, indicating that such functions may be prevalent in the arms race between plants and their attackers. *Fusarium* species are responsible for the disease *Fusarium* head blight (FHB) in major small-grain cereals such as wheat, barley and oat [142], causing estimated annual yield losses of wheat in China to exceed 3.4 million tonnes for the period 2000–2018 [143], while US agriculture loss during the 1990s FHB epidemics surpassed 2.6 billion USD [144]. *Fusarium* is not only a threat to food security but also food safety due to accumulation of fungal toxins, mycotoxins, upon infection in plants. The mycotoxin deoxynivalenol (DON) is a trichothecene and virulence factor produced by some *Fusarium* species [145]. In tall wheatgrass (*Thinopyrum elongatum*), a GST named Fhb7 catalyses the GS-conjugation of DON at the C-13 position (GS-C13-DON in table 2), resulting in opening of an epoxide group, thereby reducing the overall toxicity of DON [8,146]. Bioinformatic analyses revealed that the *Fhb7* gene in tall wheatgrass results from horizontal gene transfer from endophytic *Epichloe* fungi. Consequently, no *Fhb7* homologs are found in plant genomes [8], but GS-C13-DON as well as a GS-C10-DON conjugate have also been found in *Fusarium*-infected wheat (*T. aestivum*) and oat (*Avena sativa*; figure 1*c*) [147]. While DON glutathionylation at the C-10 position is a spontaneous chemical reaction and thus does not indicate GST activity, conjugation at the C-13 position is much less prone to happen spontaneously [147], and the presence of GS-C13-DON in these cereals, therefore, suggests that plant GSTs with this glutathionylating activity do exist beyond Fhb7. So far, wheat and barley GSTs have been identified that can produce both conjugates, albeit at very low efficiencies and only demonstrated *in vitro* [148].

GST-catalysed glutathionylation in plant defence is not limited to toxins. *Agrobacterium* virulence protein VirE2, responsible for binding and transport of viral single-strand DNA (ssDNA) across the plant cytosol during *Agrobacterium* infection of rice, is glutathionylated by rice *Os*GSTU5, decreasing its ability to bind to the ssDNA [7]. Over-expression and knocking out of *Os*GSTU5 resulted in, respectively, lower and higher efficiency of *Agrobacterium* infection, demonstrating that this GST does indeed play a role in pathogen resistance.

## 9. How can glutathione-conjugates be used as clues to identify glutathione transferase functions?

As described in this review and contrary to popular belief, endogenous GS-conjugates are found in several and diverse species scattered across the plant kingdom. They are essential parts of different metabolic pathways active during plant development or induced by stress (table 2). In an increasing number of examples, GSTs have been implied in or confirmed as responsible for biosynthesis or catabolism of GS-conjugates. In several cases, these discoveries were enabled by disruption of the GS-conjugate catabolic pathways, or by stress applications, both causing accumulation of specific conjugates. Hence, a potential strategy to identify more functions of GSTs as well as GS-conjugates is to target the downstream processing, possibly in combination with a wide range of stresses. The following four pathways for GS-conjugate catabolism are identified in plants: (1) the general detoxification pathway where the GS-peptide is first cleaved by GGTs or carboxypeptidases; (2) the so far seemingly Brassicales-specific variation of this pathway where the peptide is cleaved by GGPs; (3) the reductive removal of the entire GS-moiety, catalysed by GSTLs; and (4) the glyoxalase system for detoxification of the endogenous metabolite methylglyoxal that is a side product from glycolysis. The glyoxalase system is well described across organisms and proceeds via the non-enzymatic formation of a GS-conjugate, followed by its isomerization and hydrolysis [149], but there is no evidence for involvement of GSTs (or GGTs or GGPs) in this system. The GGT- and GGP-mediated cleavage reactions in, respectively, pathways 1 and 2 both lead to production of Cys-conjugates. Such conjugates have also been identified in wild-type plants producing the corresponding GS-conjugates, e.g. in grapevine [23], hops [117,118,123], *Allium* [97] and Brassicacea species [10,12,20,96] and in the DON-detoxifying *Fusarium*-infected cereals [147]. In the case of hops [107], passion fruit [106,150] and grapevine [23], the Cys-conjugate was initially the only one detected; however, these conjugates are generally not found in much higher concentrations than the GS-conjugates [23,117,118,123]. This presumably reflects that both conjugates are intermediates in the pathways towards biosynthesis of glucosinolates/camalexin, thiol aroma compounds or further degradation via the general detoxification pathway 1, and no evidence has been presented to support the existence of Cys-transferases to introduce the sulfur atom in place of a GST or spontaneous chemical reaction with GSH. Accordingly, searching for Cys-conjugates will only rarely provide more information than the search for GS-conjugates.

Disrupting the GGT- and GGP-mediated catabolic pathways has proven an effective tool in the discovery and identification of GS-conjugates, with *At*ggt4 knockout lines accumulating oxylipin-derived conjugates [112] and ggp1 and ggp3 lines accumulating the conjugates that are intermediates in the camalexin and glucosinolate biosynthetic pathways [19,128]. These pathways are largely vacuolar, following import by tonoplast by ABC transporters [103,104]. These are amenable to inhibition by vanadate, which was used to demonstrate the function of the first discovered plant ABC transporter in vacuolar import of GS-conjugates [151]. Such inhibition may be an alternative strategy to induce accumulation of GS-conjugates, potentially allowing investigations in plants where knockout lines of GGPs, GGTs or ABC transporters are not readily available. On the other hand, in arabidopsis, grapevine and garlic, some GGTs are targeted to different cellular compartments than the vacuole [105,152,153], and GSTLs are predicted and in some cases shown to be localized to the cytosol or to plastids [13,21,75]. In these cases, until GGT- or GSTL-specific inhibitors can be identified, accumulation of their substrates for discovery and identification will rely on the generation of gene knockout plants or application of relevant stresses. Several of the known GS-conjugates were identified as the result of biotic and abiotic stress, e.g. GS-OPDA and GS-PPA_1_ in arabidopsis resulting from wound response and *P. syringae* infiltration [113], GS-hexenol in grapevine resulting from both UV stress and pathogen infection [23] and the detoxification of microbial virulence factors in wheatgrass [58] and rice [7]. Hence, it will likely be useful and sometimes necessary to combine the strategy of knocking out enzymes and transporters from GS-conjugate catabolic pathways with screening across applications of multiple stresses to induce and identify physiologically relevant GS-conjugates. This will in turn also allow for a system to identify the GSTs that are potentially involved in producing the GS-conjugates by transcriptomics or proteomics approaches, as was already successful in the studies mentioned above, and in the sections regarding oxylipins and pathogen defence. Several of the GS-conjugates mentioned in this review were identified by ligand fishing approaches, where individual GSTs are immobilized on column material that is flushed with plant extracts to identify molecules that bind to the GSTs. While this approach also provides useful clues, authors have also sometimes suspected that the bound molecules were compounds formed during extraction [140,154] and some GSTs may bind molecules in ligandin sites that are not related to the active site or enzymatic activity [100]. Hence, such approaches can be complementary, but not stand alone in the process of identifying GST substrates.

An additional challenge in the identification of GST functions is the broad substrate specificity of many glutathionylating GSTs and overlapping activities compared to most metabolic enzymes, assumed to cause the lack of evident phenotypes in single-gene knockout plants. In a proof-of-concept study of germinating sorghum grain, we found spatio-temporal correlation between the GS-conjugate of the N-recycling pathway as determined by the spatial metabolomics technology mass spectrometry imaging [155] and gene expression of its catabolizing GSTLs as determined by spatio-temporally resolved transcriptomics [139]. In addition, we identified candidate GSTs for the first step of the pathway by the correlation of their expression patterns with those of the GSTLs [139]. With the current rapid developments in spatial metabolomics and transcriptomics, in the future the combination of these technologies can provide a standardized approach for investigating large enzyme families. Co-localization of potential enzyme/product pairs will not only narrow the number of candidate enzymes but also provide an additional level of evidence for the investigated reaction/process following the guilt-by-association principle.

Such approaches may finally lead to robust conclusions in situations where the proposed GST substrates are highly reactive and autotoxic compounds prone to chemical reactions with GSH. The nature of these GST substrates challenges the traditional view of an enzyme as facilitating a reaction that would not otherwise take place. This will remain an issue in elucidation of GST functions, as will their substrate promiscuity and overlapping activities. On the other hand, this may constitute a safety system that provides broad and rapid protection to the plant cell. The large degree of duplication and further evolution of the plant-specific classes of glutathionylating GSTs, the phi and tau classes, constitute an ‘evolutionary playground’ where plant metabolism is undergoing continuous evolution. For arabidopsis, it has been shown that evolutionarily younger genes are less likely to have an assigned function [25]. The question remains whether the GST activities will ever be streamlined by evolution, or if it is in fact an evolutionary benefit to retain the detoxifying safety system alongside the more specific functions. Either way, with this review, we have shown that the functions and activities of GSTs are important and diverse, that several are or have evolved from detoxification reactions and that GS-conjugates should not be overlooked as potential clues for identification of GST functions.

## Data Availability

Chromatograms of representative plant samples in which glutathione conjugates are identified for the first time (as stated in the main document) are available through the figshare repository [156]. Supplementary material is available online [157].
